# Flavonoids, Flavonoid Subclasses, and Esophageal Cancer Risk: A Meta-Analysis of Epidemiologic Studies

**DOI:** 10.3390/nu8060350

**Published:** 2016-06-08

**Authors:** Lingling Cui, Xinxin Liu, Yalan Tian, Chen Xie, Qianwen Li, Han Cui, Changqing Sun

**Affiliations:** College of Public Health, Zhengzhou University, Zhengzhou 450001, Henan, China; cuilingling0613@163.com (L.C.); suyingliu7777@163.com (X.L.); lhk0829@163.com (Y.T.); xcdn2227@126.com (C.X.); 13526729983@163.com (Q.L.); zhuxic@126.com (H.C.)

**Keywords:** flavonoids, esophageal cancer, meta-analysis

## Abstract

Flavonoids have been suggested to play a chemopreventive role in carcinogenesis. However, the epidemiologic studies assessing dietary intake of flavonoids and esophageal cancer risk have yielded inconsistent results. This study was designed to examine the association between flavonoids, each flavonoid subclass, and the risk of esophageal cancer with a meta-analysis approach. We searched for all relevant studies with a prospective cohort or case-control study design published from January 1990 to April 2016, using PUBMED, EMBASE, and Web of Science. Pooled odds ratios (ORs) were calculated using fixed or random-effect models. In total, seven articles including 2629 cases and 481,193 non-cases were selected for the meta-analysis. Comparing the highest-intake patients with the lowest-intake patients for total flavonoids and for each flavonoid subclass, we found that anthocyanidins (OR = 0.60, 95% CI: 0.49–0.74), flavanones (OR = 0.65, 95% CI: 0.49–0.86), and flavones (OR = 0.78, 95% CI 0.64–0.95) were inversely associated with the risk of esophageal cancer. However, total flavonoids showed marginal association with esophageal cancer risk (OR = 0.78, 95% CI: 0.59–1.04). In conclusion, our study suggested that dietary intake of total flavonoids, anthocyanidins, flavanones, and flavones might reduce the risk of esophageal cancer.

## 1. Introduction

Esophageal cancer ranks as the 10th most common malignancy and the eighth most common cause of cancer-related mortality worldwide. An estimated 455,800 newly diagnosed esophageal cancer cases and a related 400,200 deaths occurred worldwide in 2012 [1]. Rates vary widely among countries, with about half of all cases occurring in China [2]. A number of risk factors have been found to be strongly associated with esophageal cancer, including age, sex, cigarette smoking, alcohol drinking, body mass index (BMI), helicobacter pylori (H.p.) infection, low intake of fruits and vegetables, and poor nutritional status [3,4,5]. Epidemiologic studies and systematic analyses have suggest that diets rich in fruits and vegetables are associated with a reduced risk of cancer, in particular digestive tract cancers, such as esophageal cancer [6,7], gastric cancer [8,9], and colorectal cancer [10].

High intake of fruits and vegetables is associated with beneficial health effects [11], and this has been attributed in part to their high content of flavonoids. Flavonoids represent one of the largest groups of plant-specific secondary metabolites, with more than 8000 different compounds described in the literature [12]. Dietary flavonoids occur ubiquitously in plant foods, such as fruits, vegetables, tea, soybean, grains, and their processed foodstuffs, and can be categorized into six major subclasses based on their structural complexity: flavonols, flavones, flavan-3-ols, flavanones, anthocyanins, and isoflavones [13]. In addition to their antioxidant properties [14], flavonoids also have antiviral, antiallergic, antiinflammatory, and antitumor activities [15,16].

Most of the cancer preventive effects of flavonoids have been shown in animal and cell culture studies [17,18,19]. Freeze-dried berries enriched in anthocyanins have been evaluated for chemopreventive potential of premalignant oral lesions [20,21,22], familial adenomatous polyposis [23,24], Barrett’s esophagus [25], and esophageal dysplastic lesions [26]. Flavopiridol is a plant-derived semisynthetic flavone, that acts as a cyclin-dependent kinase inhibitor, it has shown promising clinical activity when combined with chemotherapy for treatment of advanced solid tumors, including those that occur in gastric cancer and esophageal cancer [27,28]. To date, meta-analyses have mainly focused on dietary flavonoids and breast cancer [29], lung cancer [30], and stomach and colorectal cancer [16]. The latest meta-analysis found no significant association between dietary flavonols intake and the risk of esophageal cancer in four studies [31]. The results of published epidemiological studies are controversial because most of the studies found no significant association between dietary flavonoid intake and esophageal cancer; whereas two studies showed that flavonoids were associated with had significant reductions in the risk of esophageal cancer [32,33]. Therefore, we performed a meta-analysis of published epidemiologic studies to further assess the association between dietary flavonoid intake and esophageal cancer risk.

## 2. Materials and Methods

### 2.1. Data Sources and Search Strategy

A comprehensive literature search was conducted to assess the associations between total flavonoids, flavonoid subclasses, and esophageal cancer. Published papers were identified from three electronic databases: PUBMED [34], EMBASE [35], and Web of Science [36] for the period from January 1990 to April 2016. The following search terms were used: (flavonoids OR flavonols OR flavones OR flavanones OR flavan-3-ols OR flavanols OR anthocyanidins OR proanthocyanidins OR isoflavones) AND (esophageal OR esophagus OR oesophageal OR oesophagus) AND (neoplasm OR neoplasms OR cancer OR cancers OR carcinoma OR tumor OR tumors OR tumour OR tumours). Additionally, the references of the original literature reports and the related articles were also searched for potential complements, especially reviews and meta-analysis papers. Only full length original journal articles were considered and no attempt was made to include abstracts or unpublished studies.

### 2.2. Inclusion Criteria and Exclusion Criterion

The following inclusion criteria were used for the present meta-analysis: (1) original articles about the association between total flavonoids, flavonoid subclasses, and esophageal cancer; (2) the study design is cohort or case-control study; (3) total flavonoids or flavonoid subclasses intake was estimated by food frequency questionnaire (FFQ) and the flavonoid food composition database; (4) relative risk (RR), hazard ratio (HR), or odds ratio (OR), and corresponding 95% confidence intervals (95% CI) were available; (5) published in English.

Accordingly, the following exclusion criteria were also considered: (1) cell studies and animal studies; (2) abstracts, reviews, letters to the editor, case reports, and repeated publications; (3) data related to exposure assessment (blood/urinary levels) of total flavonoids or one of the flavonoid subclasses; (4) studies without sufficient data for estimating the OR with 95%CI; (5) published in other languages.

Originally, we included RCTs in our search criteria, but because there were no RCTs of flavonoid intervention, no RCTs were included in the present study.

### 2.3. Data Extraction

According to the Meta-Analysis of Observational Studies in Epidemiology guidelines [37], two reviewers (Lingling Cui and Yalan Tian) independently extracted the following data from each eligible study, and discrepancies were resolved by a third investigator: (1) name of the first author and publication year; (2) country of origin; (3) study design (cohort or case-control study); (4) source of control; (5) number of cases and non-cases; (6) assessment method of dietary flavonoid intake; (7) RR, HR, or OR from the most fully adjusted model for the highest *versus* the lowest flavonoid exposure and their 95% CI; (8) confounders adjusted for in multivariate analysis.

### 2.4. Statistical Analysis

Six case-control studies provided ORs and one cohort study provided HR together with their corresponding 95% CIs. Since the absolute risk of esophageal cancer was low, the OR was theoretically similar to HR [38]. Therefore, the combined ORs together with their corresponding 95% CIs were used to calculate and assess the strength of the associations between the intake of total flavonoids, intake of each flavonoid subclass, and the risk of esophageal cancer. The heterogeneity assumption was examined by a Chi-square test based on a Q-test. Generally, *I^2^* statistics of 25%, 50%, and 75% indicate low, moderate, and high levels of heterogeneity, respectively. If *p* < 0.05 and *I*^2^ > 50%, a random-effect model based on the DerSimonian and Laird method was used to calculate pooled ORs (95% CIs). Otherwise, a fixed-effect model based on the Mantel–Haenszel method was used [39]. Meta-regression and subgroup analysis were performed to explore the source of heterogeneity based on study design (case-control or cohort), pathological type (EAC, ESCC, and Mix type—which refers to both EAC and ESCC cases—were included in the study), source of control (population-based or hospital-based), geographic location (America or Europe), dietary assessment (validated FFQ or not validated FFQ), length of dietary recall (0–5 years before diagnosis or ≥5 years before diagnosis), and adjustment for energy intake (yes or no).

Potential publication bias was examined by funnel plot and Egger’s test (*p* < 0.10) [40]. The sensitivity analysis was performed by omitting one study at a time to assess the stability of the meta-analysis results. An unchanged pooled OR implied a stable result. The statistical analyses were performed using STATA version 11.0 (Stata Corporation, College Station, TX, USA). All the *p* values were for a two-sided test, and *p* < 0.05 was considered statistically significant.

## 3. Results

### 3.1. Characteristics of the Included Studies

**Figure 1:**
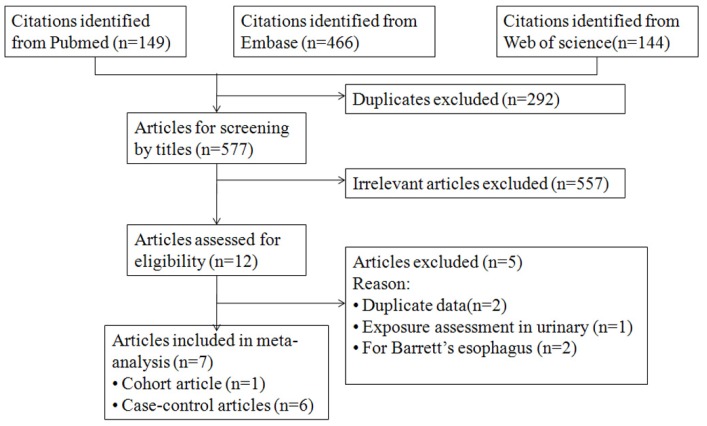
A flow diagram for selection of studies and specific reasons for exclusion from this meta-analysis.

With our search strategy, we identified 149 citations, 466 citations, and 144 citations from PUBMED, EMBASE, and Web of science, respectively. After removing 292 duplicate papers, 577 articles remained. Twelve articles were reviewed in full text after reviewing the titles and abstracts. Among them, two articles reported results for a duplicate population [41,42], one study reported urinary tea polyphenol in relation to gastric cancer and esophageal cancer [43], and another two articles reported the association between dietary flavonoid intake and Barrett’s esophagus [44,45]. As a result, seven articles reporting 12 studies including 2629 cases and 481,193 controls were selected for the meta-analysis [32,33,46,47,48,49,50]. The detailed processes of our literature search are shown in Figure 1. The main characteristics of these studies are presented in Table 1. Among them, one article is a cohort study and six studies are case–control studies. The study by Lin *et al.* only investigated the effect of three phytochemicals, including resveratrol, quercetin, and lignans on esophageal cancer [47]. Because resveratrol and lignans are not flavonoids, we only listed the result of the study by Lin *et al.* [47]*.* All of the individual studies were adjusted for a wide range of potential confounders, including age, sex, race, education, energy intake, BMI, physical activity, parity, smoking, and alcohol drinking.

### 3.2. Meta-Analysis of Flavonoids Intake and Esophageal Cancer Risk

**Figure 2:**
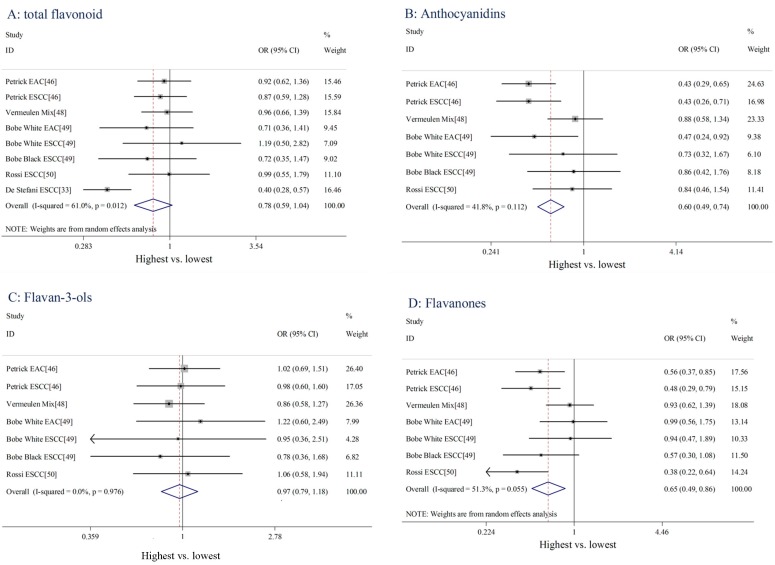

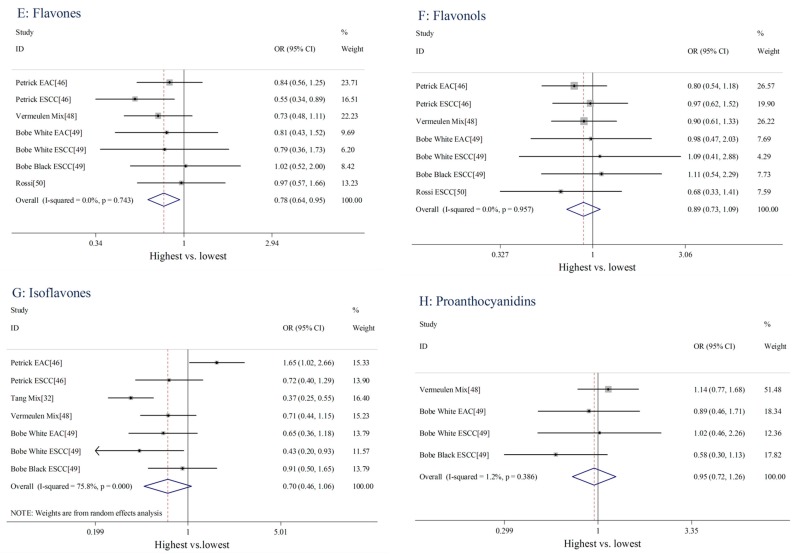
Forest plots for the association between dietary flavonoid intake and esophageal cancer risk (highest *vs.* lowest categories).

We identified eight studies of total flavonoid intake and esophageal cancer, seven studies of anthocyanidins, flavan-3-ols, flavanones, flavones, flavonols and isoflavones, four studies of proanthocyanidins and one study of lignans, quercetin, and resveratrol. We calculated the pooled ORs of esophageal cancer risk for the highest *vs.* lowest categories of total flavonoids and each flavonoid subclass, respectively. As shown in Figure 2 and Table 2, significant heterogeneity existed across studies of the total flavonoid (*I*^2^ = 61.0, *p* = 0.012), flavanones (*I*^2^ = 51.3%, *p* = 0.055), and isoflavones (*I*^2^ = 75.8%, *p* < 0.001). However no substantial heterogeneity existed across the studies of anthocyanidins (*I*^2^ = 41.8%, *p* *=* 0.112), flavan-3-ols (*I*^2^ = 0%, *p* = 0.976), flavones (*I*^2^ = 0%, *p* = 0.743) and flavonols (*I*^2^ = 0%, *p* = 0.957) and proanthocyanidins (*I*^2^ = 1.2%, *p* = 0.386). Overall, the risk of esophageal cancer significantly decreased in patients with the highest intake of anthocyanidins (OR = 0.60, 95%CI: 0.49–0.74), flavanones (OR = 0.65, 95% CI: 0.49–0.86), flavones (OR = 0.78, 95%CI: 0.64–0.95) by 40%, 35%, and 22%, respectively, compared to patients with the lowest intake of total flavonoids, anthocyanidins, flavanones, and flavones. However, marginal association of total flavonoids (OR = 0.78, 95% CI: 0.59–1.04) and no significant association of flavan-3-ols (OR = 0.97, 95% CI: 0.79–1.18), flavonols (OR = 0.89, 95% CI: 0.73–1.09), isoflavones (OR = 0.70, 95% CI: 0.46–1.06), or proanthocyanidins (OR = 0.95, 95% CI: 0.72–1.26) with esophageal cancer risk was observed.

### 3.3. Source of Heterogeneity

Substantial heterogeneity existed across studies of total flavonoid intake, and meta-regression and subgroup analysis were performed to find the source of heterogeneity. Meta-regression analysis found dietary assessment (*p* = 0.043) was the source of heterogeneity, source of control (*p* = 0.072) was a possible source of heterogeneity, and study design, pathological type (*p* = 0.860), geographic location (*p* = 0.368), period of dietary assessment (*p* = 0.850), and adjustment for energy (*p* = 0.850) showed no significant impact on between-study heterogeneity. When stratified by pathological type, source of control, geographic location, dietary assessment, length of dietary recall, and adjustment for energy intake, no statistically significant heterogeneity was found in the studies of EAC (*I*^2^ = 0%, *p* = 0.520), studies with population-based control population (*I*^2^=0%, *p* = 0.520), studies conducted in European populations (*I*^2^ = 0%, *p* = 0.931), studies using validated FFQ for flavonoid intake estimation (*I*^2^ = 0%, *p* = 0.979), studies in which dietary intake was recalled at least five years before diagnosis (*I*^2^ = 0%, *p* = 0.604), and studies without adjustment for energy intake (*I*^2^ = 0%, *p* = 0.604).

### 3.4. Sensitivity Analysis and Publication Bias

**Figure 3:**
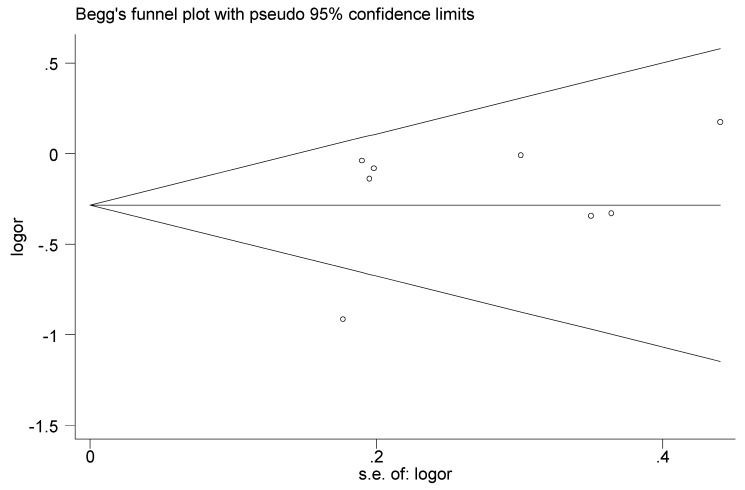
Funnel plots of total flavonoids intake and the risk of esophageal cancer.

**Figure A1:**
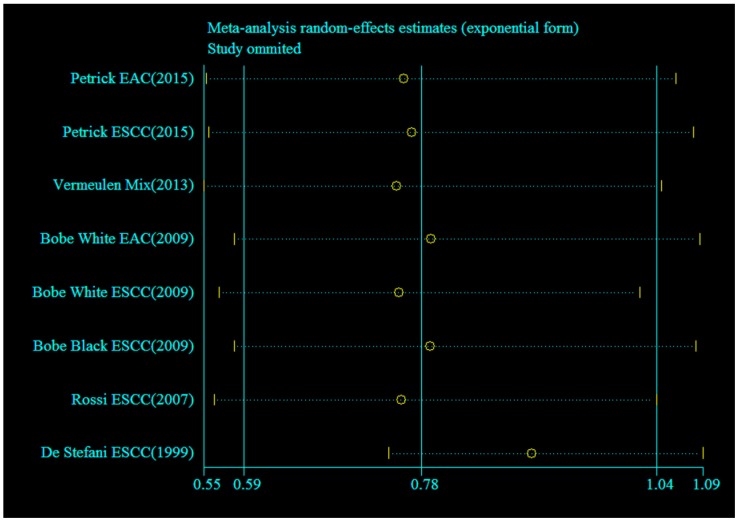
Sensitivity analysis which one study was removed at a time was performed to evaluate the stability of the results.

Sensitivity analysis suggested that no individual study significantly affected the pooled OR, which indicated that our results were statistically robust (Figure A1). Egger’s test showed no evidence of significant publication bias for the meta-analysis on the association between total flavonoids and esophageal cancer risk in eight studies (*p* = 0.454). Funnel plots were provided in Figure 3.

## 4. Discussion

Flavonoids are a large group of ubiquitous polyphenolic secondary metabolites in plants with a wide range of properties, including a widely reported anti-cancer effect. It is hypothesized that flavonoids can reduce the risk of cancer by reducing oxidative damage [51], quenching free radicals, inducing apoptosis, cell cycle arrest, and interfering with other signaling pathways [52]. As the first comprehensive meta-analysis, our study suggested a marginal association between total flavonoid intake and the risk of esophageal cancer, and also found a significant inverse association between the intake of flavonoid subclasses (anthocyanidins, flavanones, and flavones) and the risk of esophageal cancer. The findings might provide useful insight and evidence for esophageal cancer prevention, especially in areas with a relatively high incidence of esophageal cancer. Many epidemiological studies and several published meta-analyses [6,7] have found that the intake of vegetables and fruit may significantly reduce the risk of esophageal cancer; these studies have surmised that the increased flavonoid content associated with the higher intake of vegetables and fruit might be the possible mechanism underlying the observed risk reduction.

To date, the association between dietary flavonoid intake and various cancer risks has remained inconclusive. The published meta-analyses have found that total dietary flavonoid intake was not associated with a reduced risk of colorectal or stomach cancer, and dietary intake of flavonols, flavan-3-ols, anthocyanidins, and proanthocyanidins showed a significant inverse association with colorectal cancer risk. However, a significant association was found only between flavonols and stomach cancer risk based on cohort studies [16]. The latest publication found that high intake of dietary flavonols was significantly related to a reduced risk of gastric cancer, not of esophageal cancer [31], which was same as our result of seven studies from four articles. Therefore, more epidemiological research should be conducted to evaluate the effect of dietary flavonols on esophageal cancer risk. The combined results from a meta-analysis indicated a statistically significant inverse association between flavonoids intake and risk of lung cancer [30]. Previous meta-analysis showed that dietary intake of flavonols and flavones, but not of other flavonoid subclasses or total flavonoids, was associated with a decreased risk of breast cancer, especially among post-menopausal women [53]. However, a recent meta-analysis suggested that soy isoflavone intake has a protective effect against breast cancer for both pre- and post-menopausal women [54]. Another meta-analysis found dietary isoflavone intake is not statistically significantly associated with breast cancer risk in a multiethnic cohort study [29]. Our result found isoflavone intake marginally decreased the risk of esophageal cancer from seven studies in four publications, but will require more studies to confirm the association.

The present meta-analysis indicated that anthocyanidins intake was significantly reduced the risk of esophageal cancer. These compounds provide pigmentation to many fruits and vegetables, such as berries, red grapes, purple sweet potato, and red cabbages [55]. As black raspberry components, anthocyanidins could inhibit proliferation, induce apoptosis, and modulate gene expression in rat esophageal epithelial cells [56]. A previous publication reported that the anthocyanidins in freeze-dried black raspberries have chemopreventive potential in reducing NMBA tumorigenesis in the esophagus in F344 rats, via their inhibitory effects on genes associated with inflammation [57,58]. *In vitro*, flavones (luteolin, apigenin, chrysin) were able to induce cytotoxicity in KYSE-510 cells in a dose- and time-dependent manner. The effect of flavanones on esophageal cancer has been only scarcely investigated previously. Therefore, more research on the mechanism of flavones and flavanones on the development of esophageal cancer should be carried on in future.

Given that heterogeneity existed across studies of the total flavonoid intake, meta-regression and subgroup analysis was performed to find the source of heterogeneity. Meta-regression analysis found dietary assessment was the source of heterogeneity, source of control was a possible source of heterogeneity. When stratified by pathological type, source of control, geographic location, dietary assessment, length of dietary recall, and adjustment for energy intake, the heterogeneity decreased; no significant heterogeneity was found in the studies of EAC, studies with population-based controls, studies conducted in European populations, studies using validated FFQ for flavonoids intake estimation, studies in which dietary intake were recalled for at least five years before diagnosis, and studies without adjustment for energy intake.

In our view, the present meta-analysis has several advantages. First, this study is the first comprehensive meta-analysis evaluating the potential association between dietary intake of flavonoids and esophageal cancer risk. Second, all of the individual studies were adjusted for a wide range of potential confounders, including age, sex, race, education, energy intake, BMI, physical activity, parity, smoking, and alcohol drinking. Third, in addition to the estimation between total flavonoids and esophageal cancer risk, the association between subclasses of flavonoids and esophageal cancer risk was also assessed. However, several limitations should be also taken into consideration. First, the number of studies involved in the meta-analysis was not large enough. Second, this meta-analysis had regional restrictions, as all of the included studies were conducted in western countries. Third, most of the eligible studies were case–control studies, in which the information recorded about past dietary assessment would have been prone to recall bias. Selection bias often occurs if the recruited cases or controls are systematically different from the population of people who they are intended to represent in case-control studies. Fourth, the included cohort study conducted in 10 Europe countries used country specific dietary questionnaires, which might influence the results. Finally, we did not conduct the dose-response analysis of the relationships for the intake of flavonoids and esophageal risk for the irregularity and incomplete data of flavonoid intake.

## 5. Conclusions

In conclusion, the present study indicated that dietary intake of total flavonoids, anthocyanidins, flavanones, and flavones—but not intake of other flavonoid subclasses—was associated with a decreased risk of esophageal cancer. Therefore, more carefully designed studies, especially cohort studies or randomized controlled trials, should be conducted to investigate the association of dietary flavonoids intake with the risk of esophageal cancer worldwide.

## Figures and Tables

**Table 1:** Characteristics of the included studies of dietary flavonoid intake and risk of esophageal cancer.

Author, Year	Country	Study-Design	Source of Control	Dietary Assessment	Participants (Cases)	Total/Subclasses of Flavonoid	Comparison	HR or OR (95% CI)	Adjustment for Covariates
Petrick, 2015 [46]	USA	Case-control	PB	Validated FFQ-104 items	1127 (465)	Total flavonoids	≥217.36 *vs.* 0–63.8 mg/day	0.92 (0.63, 1.37) for EAC	Age, sex, race, geographic centre, cigarette smoking, and dietary energy intake
								0.87 (0.53, 1.41) for ESCC	
						Anthocyanidins	≥18.48 *vs.* 0–7.21 mg/day	0.43 (0.29, 0.66) for EAC	
								0.43 (0.26, 0.70) for ESCC	
						Flavan-3-ols	≥130.7 *vs.* 0–10.29 mg/day	1.02 (0.69, 1.51) for EAC	
								0.98 (0.60, 1.59) for ESCC	
						Flavanones	≥49.53 *vs.* 0–11.57 mg/day	0.56 (0.37, 0.85) for EAC	
								0.48 (0.29, 0.78) for ESCC	
						Flavones	≥2.63 *vs.* 0–1.29 mg/day	0.84 (0.56, 1.25) for EAC	
								0.55 (0.34, 0.89) for ESCC	
						Flavonols	≥17.8 *vs.* 0–8.31 mg/day	0.80 (0.54, 1.18) for EAC	
								0.97 (0.62, 1.53) for ESCC	
						Isoflavones	≥0.60 *vs.* 0–0.27 mg/day	1.65 (1.02, 2.65) for EAC	
								0.72 (0.40, 1.29) for ESCC	
						Lignans	≥0.083 *vs.* 0–0.045 mg/day	0.75 (0.49, 1.13) for EAC	
								0.38 (0.23, 0.63) for ESCC	
Tang, 2015 [32]	China	Case-control	HB	Validated FFQ-137 items	739 (359)	Isoflavones	>26.0 *vs.* <8.0 mg/day	0.37 (0.25–0.55)	Age, gender, education level, BMI, total energy intake (kJ/d), tobacco smoking, alcohol drinking, and family history of cancer
Lin, 2014 [47]	Sweden	Case-control	PB	Validated FFQ-36 items	1407 (601)	Resveratrol, quercetin, and lignans	Q5 *vs.* Q1	0.24 (0.12–0.49) for EAC 0.31 (0.15–0.65) for ESCC 0.42 (0.26–0.67) for JAC	Age, sex, energy, educational level, smoking, alcohol consumption, BMI, physical activity, reflux, and Helicobacter pylori infection.
Vermeulen, 2013 [48]	23 centers in 10 European countries.	Cohort	PB	Validated FFQ 1877 items	477,312 (341)	Total flavonoids	Q4 *vs.* Q1	0.96 (0.66–1.39)	Center, age, sex, energy intake, BMI, smoking intensity, educational level, physical activity, alcohol, red and processed meat intake, fiber, vitamin C, and carotenoids
						Flavanols		0.65 (0.66–1.38)	
						Flavan-3-ol		0.86 (0.58–1.27)	
						Proanthocyanidins		1.14 (0.77–1.68)	
						Theaflavins		0.76 (0.53–1.10)	
						Anthocyanidins		0.88 (0.58–1.35)	
						Flavonols		0.90 (0.61–1.34)	
						Flavanones		0.93 (0.62–1.38)	
						Flavones		0.73 (0.48–1.10)	
						Isoflavones		0.71 (0.44–1.16)	
Bobe, 2009 [49]	United States	Case-control	PB	Not validated FFQ-57 items	1728 (493)	Total Flavonoids	>107 *vs.* <43.0 mg/1000 kcal	0.71 (0.36–1.42) for White EAC	Smoking duration and intensity, geographical area, age, BMI, hot tea consumption, hard liquor consumption, beer consumption, “moonshine” consumption (only for black men), red wine consumption, white wine consumption (except for ESCC in white men), caloric intake, education (only for black men), and income.
								1.19 (0.50–2.81) for White ESCC	
								0.72 (0.35–1.46) for Black ESCC	
						Anthocyanidins	>4.73 *vs.* <1.45 mg/1000 kcal	0.47 (0.24–0.91) for White EAC	
								0.73 (0.32–1.67) for White ESCC	
								0.86 (0.42–1.75) for Black ESCC	
						Flavan-3-ols	>60.6 *vs.* <10.3 mg/1000 kcal	1.22 (0.60–2.49) for White EAC	
								0.95 (0.36–2.52) for White ESCC	
								0.78 (0.36–1.68) for Black ESCC	
						Flavanones	>26.2 *vs.* <9.3 mg/1000 kcal	0.99 (0.56–1.75) for White EAC	
								0.94 (0.47–1.90) for White ESCC	
								0.57 (0.30–1.08) for Black ESCC	
						Flavones	>4.41 *vs.* <2.08 mg/1000 kcal	0.81 (0.43–1.51) for White EAC	
								0.79 (0.36–1.73) for White ESCC	
								1.02 (0.52–2.00) for Black ESCC	
						Flavonols	>15.9 *vs.* <6.89 mg/1000 kcal	0.98 (0.47–2.01) for White EAC	
								1.09 (0.41–2.87) for White ESCC	
								1.11 (0.54–2.30) for Black ESCC	
						Isoflavonoids	>0.019 *vs.* <0.005 mg/1000 kcal	0.65 (0.36–1.18) for White EAC	
								0.43 (0.20–0.93) for White ESCC	
								0.91 (0.50–1.64) for Black ESCC	
						Proanthocyanidins	>272 *vs.* 45.5 mg/1000 kcal	0.89 (0.46–1.70) for White EAC	
								1.02 (0.46–2.26) for White ESCC	
								0.58 (0.30–1.13) for Black ESCC	
Rossi, 2007 [50]	Italy	Case-control	HB	Validated FFQ-78 items,	1047 (304)	Total Flavonoids	Q5 *vs.* Q1	0.99 (0.55–1.79)	Age, sex, study centre, education, alcohol consumption, tobacco smoking, BMI, and energy intake.
						Anthocyanidins		0.84 (0.46–1.54)	
						Flavan-3-ols		1.06 (0.58–1.94)	
						Flavanones		0.38 (0.23–0.66)	
						Flavones		0.97 (0.57–1.67)	
						Flavonols		0.68 (0.38–1.64)	
De Stefani, 1999 [33]	Uruguay	Case-control	HB	Not validated FFQ-64 items	459 (66)	Flavonoids	Q3 *vs.* Q1	0.4 (0.3–0.6)	Age, sex, residence, urban/rural, education, BMI, tobacco smoking, alcohol, and energy

EAC: esophageal adenocarcinoma, ESCC: esophageal squamous cell carcinoma, FFQ: food-frequency questionnaire.

**Table 2:** Meta-analysis of risk estimates of flavonoids intake (highest *versus* lowest) and esophageal cancer risk.

Subgroups	No. of Studies	No. of Cases	Pooled ORs (95% CI)	*p*	Heterogeneity Test		
					Chi-Square	*I*^2^ (%)	*p_het_*
**All studies**	8	1673	0.78 (0.59–1.04)	0.088	17.95	61.0	0.012
**Subclass of flavonoids**							
Anthocyanidins	7	1607	0.60 (0.49–0.74)	<0.001	10.31	41.8	0.112
Flavan-3-ols	7	1607	0.97 (0.79–1.18)	0.735	1.22	0	0.976
Flavanones	7	1607	0.65 (0.49–0.86)	0.002	12.33	51.3	0.055
Flavones	7	1607	0.78 (0.64–0.95)	0.013	3.51	0	0.743
Flavonols	7	1607	0.89 (0.73–1.09)	0.276	1.54	0	0.957
Isoflavones	7	1662	0.70 (0.46–1.06)	0.093	24.77	75.8	<0.001
Proanthocyanidins	4	838	0.95 (0.72–1.26)	0.734	3.04	1.2	0.386
**Study design**							
Cohort	1	345	0.96 (0.66–1.39)	0.830	N/A	N/A	N/A
Case-control	7	1328	0.76 (0.55–1.04)	0.088	15.92	62.3	0.014
**Pathological type**							
EAC	2	435	0.86 (0.62–1.21)	0.396	0.41	0	0.520
ESCC	5	893	0.74 (0.48–1.15)	0.051	13.68	70.8	0.008
Mix type	1	345	0.96 (0.66–1.39)	0.830	N/A	N/A	N/A
**Source of control**							
Hospital-based	2	370	0.61 (0.25–1.48)	0.273	6.74	85.2	0.009
Population-based	6	1303	0.89 (0.74–1.09)	0.260	1.39	0	0.926
**Geographic locations**							
Europe	2	649	0.97 (0.71–1.33)	0.842	0.01	0	0.931
America	6	1024	0.73 (0.51–1.04)	0.080	14.57	65.7	0.012
**Dietary assessment**							
Validated FFQ	4	1114	0.93 (0.75–1.14)	0.461	0.19	0	0.979
Not Validated FFQ	4	559	0.64 (0.39–1.04)	0.070	7.31	59.0	0.063
**Length of dietary recall**							
0–5 years before diagnosis	5	1180	0.78 (0.54–1.12)	0.178	16.81	76.2	0.002
≥5 years before diagnosis	3	493	0.81 (0.53–1.25)	0.338	1.01	0	0.604
**Adjustment for energy intake**							
Yes	5	1180	0.78 (0.54–1.12)	0.178	16.81	76.2	0.002
No	3	493	0.81 (0.53–1.25)	0.338	1.01	0	0.604

EAC: esophageal adenocarcinoma, ESCC: esophageal squamous cell carcinoma, FFQ: food-frequency questionnaire, N/A: Not applicable.
